# Feasibility of group cognitive behavioural therapy for insomnia (CBT-I) in bipolar disorder

**DOI:** 10.1192/j.eurpsy.2021.518

**Published:** 2021-08-13

**Authors:** O. Swagemakers, A. Nugter, F. Engelsbel, P. Schulte

**Affiliations:** Research Department, GGZ Noord-Holland-Noord, Heerhugowaard, Netherlands

**Keywords:** bipolar disorder, Insomnia, cognitive behavior therapy, group therapy

## Abstract

**Introduction:**

Euthymic patients with bipolar I and II disorder (BD) often have comorbid insomnia, which is associated with worse outcome. Cognitive behavioral therapy for insomnia (CBT-I) is rarely offered to this population, though preliminary research indicates CBT-I to be safe and helpful to improve sleep and mood stability.

**Objectives:**

The present study investigates if CBT-I for euthymic BD patients is feasible and acceptable when offered in a group format.

**Methods:**

14 euthymic bipolar disorder I or II participants participated in a 7-session group CBT-I with BD-specific modifications (CBT-I-BD), preceded by one individual session. Feasibility and acceptability were assessed by recruitment, treatment drop-out and participants’ and therapists’ evaluations, while sleep quality, mood and sleep medication were assessed at baseline, end of treatment, 3 and 6 months later.

**Results:**

31 of 539 patients with bipolar disorder were referred, 14 were included and one dropped out of treatment. Group CBT-I-BD was acceptable as shown by high session attendance and good homework compliance. Participants highly appreciated the treatment, the group format and learning effect. Insomnia severity decreased significantly between baseline and post-treatment. Group CBT-I-BD did not cause mood episodes during treatment and although not requested, the total number of nights with sleep medication decreased.

**Conclusions:**

Group CBT-I-BD seems to be a feasible, acceptable and therefore viable treatment for euthymic patients with bipolar disorder suffering from persistent insomnia. The small sample size, resulting in small CBT-I-BD groups was a main limitation of the study.

